# A Further Contribution to the Study of Sagittamide A: Synthesis of a Pivotal Intermediate Belonging to a Rare L-Series

**DOI:** 10.3390/molecules17077709

**Published:** 2012-06-25

**Authors:** Anne Humbert, Karen Plé, Dominique Harakat, Agathe Martinez, Arnaud Haudrechy

**Affiliations:** Institut de Chimie Moléculaire de Reims, UMR CNRS 7312, Université de Reims, BP 1039, F-51687 REIMS Cedex, France; Email: anne.bak@hotmail.fr (A.H.); karen.ple@univ-reims.fr (K.P.); dominique.harakat@univ-reims.fr (D.H.); agathe.martinez@univ-reims.fr (A.M.)

**Keywords:** sagittamide, L-altrose, L-talose, epoxide opening, asymmetric dihydroxylation

## Abstract

A key saggitamide intermediate corresponding to a rare sugar framework has been obtained. This approach should help to establish the overall configuration of more complex structures of the sagittamide family.

## 1. Introduction

**Figure 1:**
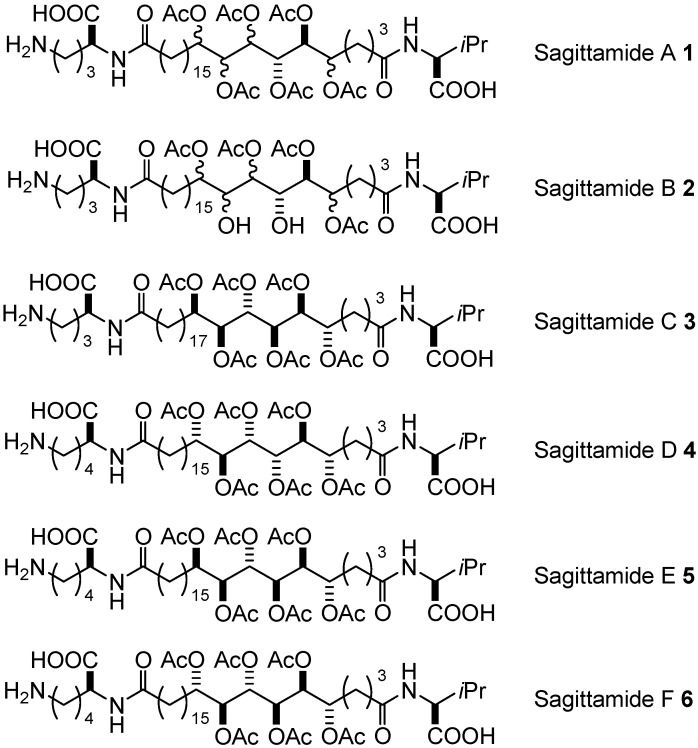
Structures proposed for Sagittamides A–F **1**–**6**.

Sagittamides A–F (**1**–**6**, Figure 1) are unprecedented marine natural products isolated from an unidentified tropical didemnid tunicate collected in Micronesia and described by Molinski [1,2]. These compounds are characterized by an α,ωw-dicarboxylic acid (C-26 or C-28) that is acylated at each terminus by a different amino acid (L-ornithine or L-lysine and L-valine), and contain a stereocluster of contiguous polyacetoxy 5,6,7,8,9,10-hexaols. After a first prediction of the stereochemistry of Sagittamide A **1** using a detailed ^1^H-NMR analysis of the ^3^*J*_H,H_ profiles [3], a second publication gave another stereochemical attribution, discussing a similar approach but reaching different conclusions (with 2D heteronuclear NMR experiments) [4], and finally a third publication extended the uncertainty, describing three other structures (only **1a** being compatible with Residual Dipolar Coupling Enhanced NMR) [5].

**Figure 2:**
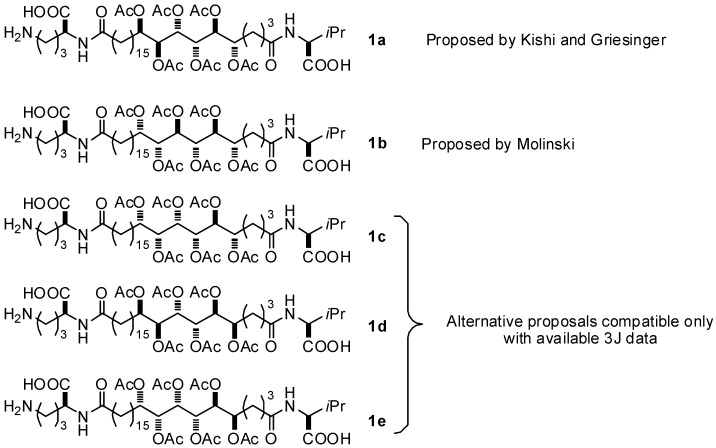
Structures proposed for Sagittamide A **1**.

When regarding the exact configuration of Sagittamides A and B (**1** and **2**, Figure 1), two points have been firmly established: (a) the configuration is *S* in positions 6 and 7; (b) There is a clear *syn* relationship between positions 9 and 10 [5]. Thus, the five possible structures proposed for Sagittamide A **1** are described in Figure 2.

**Figure 3:**
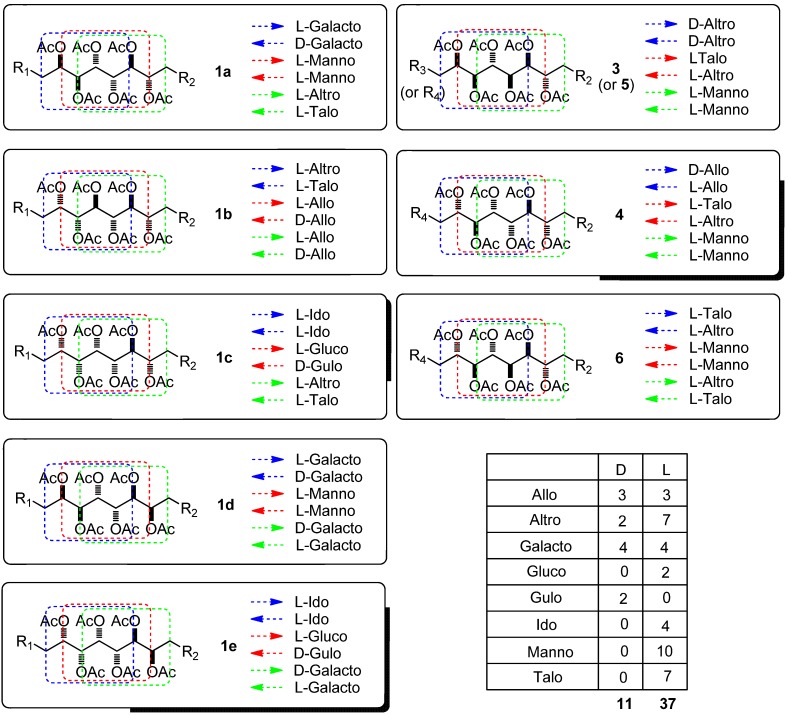
Use of the Quiral program for the study of carbohydrate backbones in the central stereoclusters of Sagittamides **A**–**F**.

Using the Quiral program developed in our laboratory [6,7], which analyzes the 3D structure of a target organic molecule to find which sugar(s) can be used as a starting material for its synthesis, a careful study of the configurations for the central stereocluster of sagittamide A **1** (and related **2**) was performed. For each possible structure (compounds **1a**–**e**, **3**–**6**), three groups of six consecutive carbons were analyzed, and their similarity to a known sugar identified in both directions (left to right and right to left, see Figure 3). The large majority of the proposed structures involve L-sugars, as seen in the summary table.

**Figure 4:**
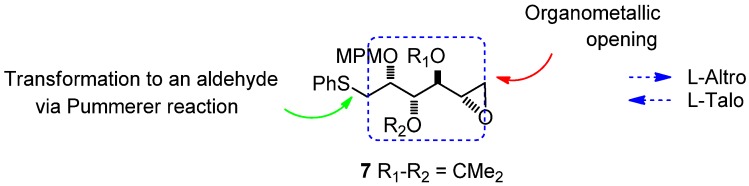
Targeted structure **7**.

Of the nine possible carbohydrate structures shown in Scheme 3, the rare sugar couple L-altrose/L-talose can be seen in seven cases (**1a**–**c**, **3**–**6**). It thus appeared feasible to start from this backbone in order to access the greatest number of structures, with any missing targets being obtained by a Mitsunobu approach. Our research was focused on finding a controlled access to the diversely protected structure **7** (Figure 4). 

On the left-hand side of the target structure **7**, the thiophenyl group is an aldehyde surrogate through a Pummerer transformation, and on the right-hand side, the epoxide could be selectively opened by diverse organometallic species.

## 2. Results and Discussion

**Scheme 1:**
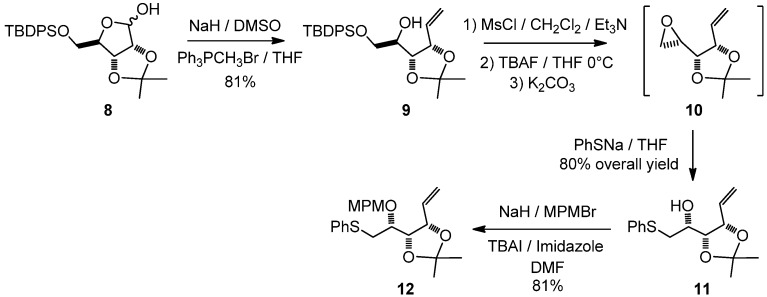
Synthesis of the alkene derivative **12**.

The protected derivative **12** was easily obtained after several straightforward reactions of the known D-ribose derivative **8** (Scheme 1) [8]. After a Wittig reaction on the lactol moiety in the presence of DMSO, the previously described open carbohydrate **9** [9] was obtained in 81% yield. Noteworthy, the use of potassium *tert*-butoxide in THF, as described in the literature [9], resulted in a substantial loss of the TBDPS group. After several unsuccessful Mitsunobu attempts under various conditions (DEAD or DIAD as activators, toluene or THF as a solvent, *p*NO_2_PhCOOH or ClCH_2_COOH as nucleophilic moieties) [10,11,12], and inspired by some of our preliminary results [13,14,15], the necessary inversion of configuration occurred very cleanly under mesylate activation, followed by an *in situ* formation of the known epoxide **10** [16]. The direct opening of this epoxide with sodium phenylsulfide offered an efficient access to the expected derivative **11** [17]. Finally, in order to install the left-hand side of our target, the free hydroxy function was protected with a MPM group to form **12**. This procedure was clean and reproducible on a multigram scale.

**Scheme 2:**
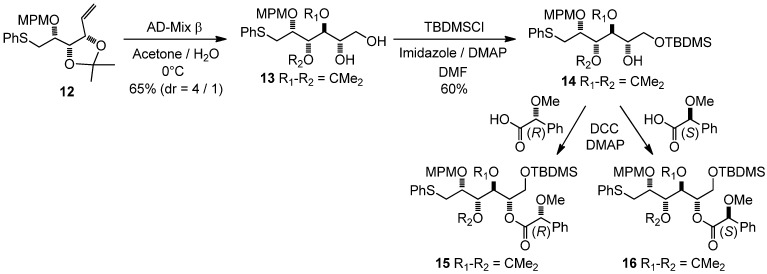
Selective osmylation in the presence of a sulfur moiety and formation of MPA esters.

Not surprisingly, the controlled oxidation of alkene **12** with one equivalent of MCPBA in CH_2_Cl_2_ very cleanly gave the corresponding sulfoxide in 84% yield. An obvious way to finalize the synthesis involved the selective osmylation of the derivative **12**. The use of osmium tetroxide (OsO_4_) seemed unsuitable because of the concurrent reactivity of the sulfur. However, precedents in the literature were found in which an AD-Mix type approach was effective for this transformation [18,19,20,21,22]. Thus, after a first attempt in a *t*BuOH/water mixture at 0 °C that gave no reaction, the use of AD-Mix b in acetone/water gave the *S* diastereoisomer as the major one, in 65% overall yield (d.r. = 80:20) with 11% of recovered starting material (Scheme 2). Interestingly, the use of AD-Mix α gave the same major diastereoisomer, with a better diastereoselectivity (7.3:1), however in lower yield (54% overall yield, with 20% of recovered starting material). This observation was quite surprising, even if a similar case has previously been reported in the literature [23]. 

**Figure 5:**
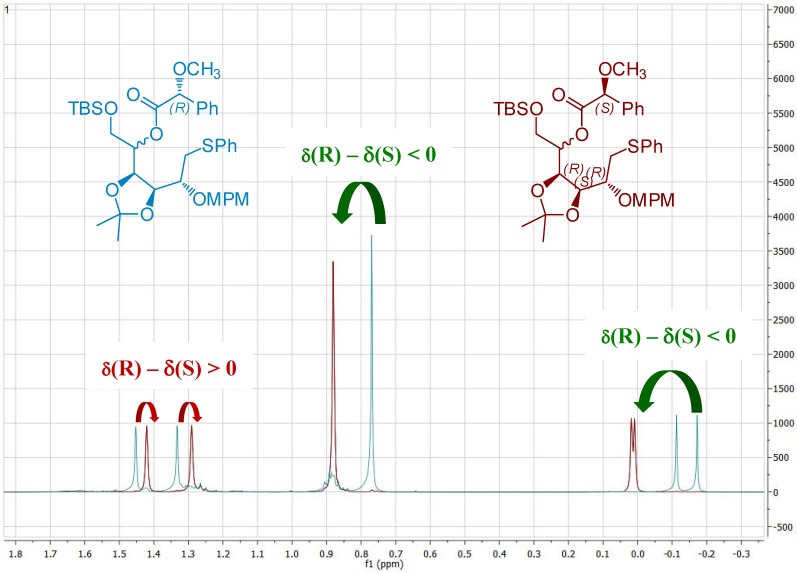
Comparison of selected ^1^H-NMR spectra of MPA ester derivatives of **14**.

After protection of the primary hydroxyl group with a TBDMS ether, the secondary alcohol **14** was esterified with (*R*) and (*S*)-MPA moieties, in order to prove the induced stereoselectivity (Figure 5) [24].

**Scheme 3:**
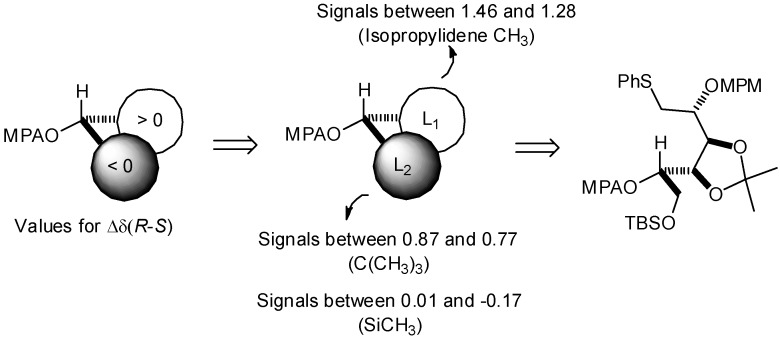
General scheme for the assignment of *R* or *S* configuration.

**Scheme 4:**
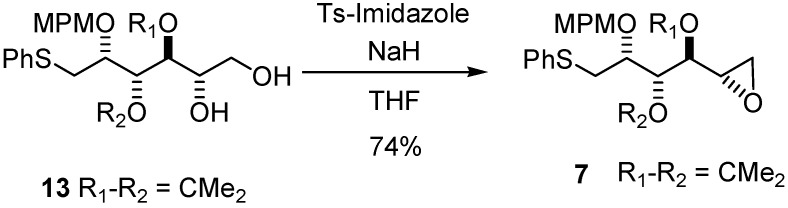
Formation of target compound **7**.

With this method, comparison of the chemical shifts of the signals due to protons in L_1_ and L_2_ in both the *R* and the *S* derivatives and calculation of the corresponding differences expressed as Δδ*R*/*S*, helps to assign the *S* configuration (Scheme 3). Finally, the desired epoxide **7** was obtained after selective intermediate tosylate activation, followed by a basic cyclization (Scheme 4). In our future synthesis of sagittamide-type structures, this epoxide could be directly opened as in one of our previously described transformations (**10** to **11**).

## 3. Experimental

### 3.1. General

All reactions were performed under argon with magnetic stirring unless otherwise specified. Dry solvents were used in all experiments. Thin layer chromatography was performed on E. Merck pre-coated 60 F_254_ plates and compounds were observed by UV or by charring the plates with an acidic anisaldehyde system. Flash chromatography was performed on an Armen SpotFlash with silica gel 60 (particle size 15–40 mm). Optical rotations were measured on a Perkin-Elmer 341 polarimeter at 20 °C. Electrospray ionization mass spectrometry experiments (MS and HRMS) were obtained on a hybrid tandem quadrupole/time-of-flight (Q-TOF) instrument, equipped with a pneumatically assisted electrospray (Z-spray) ion source (Micromass, Manchester, UK) operated in positive mode (EV = 30V, 80 °C, injection flow 5 mL/min). NMR spectra were recorded on Bruker 600, 500 or 250 MHz spectrometer. Chemical shifts were measured in δ (ppm) and coupling constants J in Hz (solvent peak reference: δ = 7.27 for ^1^H, 77.0 for ^13^C). Multiplicities are indicated by s (singlet), d (doublet), t (triplet), q (quartet), m (multiplet) or br (broad). Chemical shifts (δ) reported are referred to internal tetramethylsilane. FT-IR spectra were recorded on Nicolet Avatar 320 FT-IR as films. Elemental analyses were performed with a Thermo Flash EA 1112 Series. 

*1,2-Dideoxy-6-O-(tert-butyldiphenylsilyloxy)-3,4-di-O-isopropylidene-D-ribo-hex-1-enitol* (**9**)


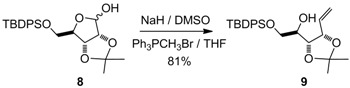


A sodium hydride suspension in mineral oil (1.16 g, 28.9 mmol) was washed three times with *n*-hexane and then suspended in DMSO (15.9 mL). After heating to 68 °C for 45 min, the grey-green mixture was transferred via syringe to a suspension of methyltriphenylphosphonium bromide (10.33 g, 28.9 mmol, co-evaporated with toluene before use) in dry THF (110 mL). After stirring for 10 min, a solution of lactol **8** (3 g, 7.23 mmol) in dry THF (50 mL) was transferred via a cannula. The yellow mixture was stirred at room temperature for 16 h and then poured into ice water (200 mL). After extraction with diethyl ether (3 × 500 mL), the combined organic phases were washed with a saturated NaCl solution (50 mL). The organic phase was dried with MgSO_4_, and after filtration and evaporation to dryness, the residue was purified by column chromatography to give **9** (2.17 g, 5.1 mmol, 81%) as a slightly yellow oil. [α]^20^_D_ = −0.4° (C = 1.04, CHCl_3_); ^1^H-NMR (CDCl_3_): δ 7.66–7.77 (m, 4 H, Ar), 7.34–7.51 (m, 6 H, Ar), 6.04 (ddd, 1 H, *J*_2,1trans_ 17.1 Hz, *J*_2,1cis _10.4 Hz, *J*_2,3_ 6.7 Hz, H-2), 5.44 (d, 1 H, *J*_1trans,2_ 17.1 Hz, H-1trans), 5.30 (d, 1 H, *J*_1cis,2 _10.4 Hz, H-1cis), 4.73 (t, 1 H, *J*_3,2 _6.7 Hz, *J*_3,4 _6.7 Hz, H-3), 4.17 (dt, 1 H, *J*_4,5_ 13.7 Hz, *J*_4,3_ 6.7 Hz, H-4), 3.87 (m, 2 H, H-6), 3.67-3.80 (m, 1 H, H-5), 2.65 (br s, 1 H, OH), 1.41 (s, 3 H, 1 C*H_3_*), 1.37 (s, 3 H, 1 C*H_3_*), 1.10 (s, 9 H, Si-(CH_3_)_3_); ^13^C-NMR (CDCl_3_): δ 136.1 (C_ortho_ or C_meta_), 136.0 (C_ortho_ or C_meta_), 134.5 (C-2), 133.5 (Cq_Ar_), 133.5 (Cq_Ar_), 130.3 (C_para_), 128.3 (C_Ar_), 128.2 (C_Ar_), 118.1 (C-1), 109.2 (Cq isopropylidene), 79.2 (C-3), 77.9 (C-4), 70.3 (C-5), 65.7 (C-6), 28.2 (CH_3_ isopropylidene), 27.3 (C(CH_3_)_3_), 25.9 (CH_3_ isopropylidene), 19.8 (C(CH_3_)_3_); IR (neat) *v*_max_: 3564, 3481, 2934, 2859, 1429, 1218, 1114, 1059, 704 cm^–1^; HRMS (*m/z*, ESI) calculated for C_25_H_34_O_4_NaSi: (M+Na) = 449.2124 (calculated), 449.2120 (found). 

*1,2-Dideoxy-3,4-di-O-isopropylidene-6-S-phenylthio-L-lyxo-hex-1-enitol* (**11**)


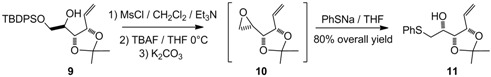


Triethylamine (4.8 mL, 34.5 mmol) followed by methanesulfonyl chloride (1.16 mL, 15 mmol) were added drop-wise to a solution of compound **9** (4.89 g, 11.5 mmol) in methylene chloride (70 mL) at 0 °C. After stirring for 16 h at room temperature, the mixture was diluted with diethyl ether (300 mL), washed with a saturated ammonium chloride solution (50 mL), and then with a saturated sodium chloride solution (50 mL). The organic phase was dried with MgSO_4_, filtered and evaporated to dryness. Dry THF (110 mL) was added and reaction mixture was cooled to 0 °C. A tetra-*n*-butyl ammonium fluoride solution (1 M in THF; 23 mL) was added drop-wise, and the reaction mixture was stirred at room temperature for 3 h. After addition of K_2_CO_3_ (2.8 g, 20 mmol) and stirring for 16 h, the reaction mixture was diluted with water (25 mL) and extracted with diethyl ether (3 × 300 mL). The combined organic phases were dried with MgSO_4_, filtered and evaporated to dryness. The crude epoxide **10** was used in the next step without further purification. Dry THF (50 mL) was then added and the reaction mixture was cooled to 0 °C, and a THF solution of sodium thiophenoxide was added [prepared by addition of thiophenol (2.94 mL, 28.8 mmol) to a suspension of sodium (1.15 g, 28.8 mmol) in dry THF (50 mL)]. The reaction was stirred at room temperature for 16 h. The crude mixture was then slowly quenched with methanol (5 mL), a half-saturated ammonium chloride solution (30 mL) was added and the aqueous layer was extracted with diethyl ether (3 × 300 mL). The combined organic phases were dried with MgSO_4_, filtered and evaporated, and the residue purified by column chromatography to give **11** (2.56 g, 9.1 mmol, 80%) as a colorless oil. [α]^20^_D_ = +27.3° (C = 1.04, CHCl_3_); ^1^H-NMR (CDCl_3_): δ 7.13–7.43 (m, 5 H, H_Ar_), 5.95 (ddd, 1 H, *J*_2,1trans_ 17.4 Hz, *J*_2,1cis _10.1 Hz, *J*_2,3_ 8.1 Hz, H-2), 5.33 (d, 1 H, H-1trans), 5.24 (d, 1 H, H-1cis), 4.59 (t, 1 H, *J*_3,4_ 7.5 Hz, H-3), 4.27 (dd, 1 H, *J*_4,5_ 3.6 Hz, H-4), 3.68 (td, 1 H, *J*_5,6_ 6.5 Hz, H-5), 3.05 (dd, 2 H, H-6), 2.44 (br s, 1 H, OH), 1.52 (s, 3 H, 1 C*H_3_*), 1.38 (s, 3 H, 1 C*H_3_*); ^13^C-NMR (CDCl_3_): δ 135.9 (Cq_Ar_), 134.2 (C-2), 130.2 (C_ortho_ or C_meta_), 129.4 (C_ortho_ or C_meta_), 126.9 (C_para_), 120.3 (C-1), 109.3 (Cq isopropylidene), 79.5 (C-3 or C-4), 78.9 (C-3 or C-4), 68.8 (C-5), 38.3 (C-6), 27.5 (CH_3_ isopropylidene), 25.3 (CH_3_ isopropylidene); IR (neat) *v*_max_: 3480, 2988, 2935, 1585, 1482, 1458, 1439, 1380, 1215, 1057, 741, 693 cm^–1^; HRMS (*m/z*, ESI) calculated for C_15_H_20_O_3_NaS: (M+Na) = 303.1031 (calculated), 303.1038 (found).

*1,2-Dideoxy-3,4-di-O-isopropylidene-5-O-(4-methoxybenzyl)-6-S-phenylthio-L-lyxo-hex-1-enitol* (**12**)


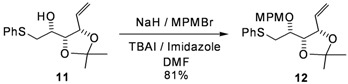


A solution of **11** (2.38 g, 8.49 mmol) in a mixture of THF/DMF (18 mL/4.5 mL) was added drop-wise to a stirring suspension of sodium hydride (560 mg, 17 mmol) in a mixture of THF/DMF (18 mL/4.5 mL) at 0 °C. After addition of tetra-*n*-butylammonium iodide (314 mg, 0.85 mmol) and imidazole (58 mg, 0.85 mmol), *p*-methoxybenzyl bromide (2.48 mL, 17 mmol) was added drop-wise and the yellow suspension was stirred at room temperature for 16 h. The reaction was quenched by the addition of methanol (5 mL) at 0 °C and after addition of water (30 mL), the reaction mixture was extracted with diethyl ether (3 × 300 mL). The combined organic phases were dried with MgSO_4_, filtered and evaporated, and the residue purified by column chromatography to give **12** (2.76 g, 6.89 mmol, 81%) as a colorless oil. [α]^20^_D_ = −2.8° (C = 1.02, CH_3_OH); ^1^H-NMR (CDCl_3_): δ 7.11–7.50 (m, 7 H, H_Ar_), 6.77–6.95 (m, 2 H, H_Ar_), 5.84 (ddd, 1 H, *J*_2,1trans_ 17.2 Hz, *J*_2,1cis _10.2 Hz, *J*_2,3_ 8.3 Hz, H-2), 5.26 (d, 1 H, H-1trans), 5.16 (d, 1 H, H-1cis), 4.59 (s, 2 H, C*H_2_*Ph), 4.50 (dd, 1 H, *J*_3,4 _6.1 Hz, H-3), 4.39 (t, 1 H, *J*_4,5_ 6.1 Hz, H-4), 3.80 (s, 3 H, OMe), 3.55 (dd, 1 H, *J*_5,6_ 11.7 Hz, H-5), 3.11 (dd, 2 H, H-6), 1.52 (s, 3 H, 1 C*H_3_*), 1.37 (s, 3 H, 1 C*H_3_*); ^13^C-NMR (CDCl_3_): δ 159.5 (Cq_ArOMe_), 136.7 (Cq_ArSPh_), 134.8 (C-2), 130.9 (Cq_ArCH2O_), 130.3 (C_Ar_), 129.8 (C_Ar_), 129.3 (C_Ar_), 126.7 (C_Ar/paraS_), 119.6 (C-1), 114.1 (C_Ar/orthoOMe_), 109.7 (Cq isopropylidene), 80.0 (C-4), 79.5 (C-3), 77.0 (C-5), 72.8 (*C*H_2_Ph), 55.7 (O*Me*), 35.5 (C-6), 28.0 (CH_3_ isopropylidene), 26.0 (CH_3_ isopropylidene); IR (neat) *v*_max_: 2986, 2935, 2837, 1613, 1614, 1248, 1038, 742, 693 cm^–1^; HRMS (*m/z*, ESI) calculated for C_23_H_28_O_4_NaS: (M+Na) = 423.1606 (calculated), 423.1601 (found).

*3,4-Di-O-isopropylidene-2-O-(4-methoxybenzyl)-1-S-phenylthio-L-altritol* (**13**)


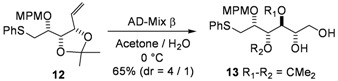


A solution of **12** (654 mg, 1.63 mmol) in a mixture of acetone/water (4 mL 1:1) was added to AD-Mix b (3 g) in a mixture of acetone/water (16 mL 1:1) at 0 °C. After stirring for 18 h, sodium sulfite (3.34 g) was added. After dilution with water (2 mL), the reaction was extracted with methylene chloride (3 × 100 mL). The combined organic phases were dried with MgSO_4_, filtered and evaporated, and the residue purified by column chromatography to give **13** (368 mg, 52%) as the major diastereoisomer, and 3,4-di-*O*-isopropylidene-2-*O*-(4-methoxybenzyl)-1-*S*-phenylthio-D-galactitol (92 mg, 13%) as the minor one, both as colorless oils (diastereoisomeric ratio 4/1), with 11% of recovered starting material. The same reaction with AD-Mix α gave **13** (336 mg, 47.5%) as the major diastereoisomer, and 3,4-di-*O*-isopropylidene-2-*O*-(4-methoxybenzyl)-1-*S*-phenylthio-D-galactitol (46 mg, 6.5%) as the minor diastereoisomer, (diastereoisomeric ratio 7.3/1), with 20% of recovered starting material.

Major diol (**13**): [α]^20^_D_ = −0.4° (C = 0.975, CH_3_OH); ^1^H-NMR (CDCl_3_): δ 7.10–7.46 (m, 7 H, H_Ar_), 6.84 (d, 2 H, *J*_ortho,meta/OMe _8.6 Hz, H_Ar_), 4.70 (d, 1 H, *J*_CHPh,CPh_ 10.9 Hz, C*H*_2_Ph), 4.52 (d, 1 H, C*H*_2_Ph), 4.41 (dd, 1 H, *J*_3,4_ 6.1 Hz, *J*_3,2_ 4.3 Hz, H-3), 4.09 (dd, 1 H, *J*_4,5_ 9.3 Hz, H-4), 3.93–4.02 (m, 1 H, H-2), 3.84–3.93 (m, 1 H, H-5), 3.73–3.82 (m, 4 H, H-6a, OMe), 3.61 (dd, 1 H, *J*_6b,6a_ 11.2 Hz, *J*_6b,5_ 5.6 Hz, H-6b), 3.42–3.56 (br s, 1 H, OH), 3.46 (dd, 1 H, *J*_1a,2_ 5.0 Hz, *J*_1a,1b_ 13.7 Hz, H-1a), 3.25 (dd, 1 H, *J*_1b,2_ 7.2 Hz, H-1b), 2.22 (br s, 1 H, OH), 1.46 (s, 3 H, 1 C*H_3_*), 1.31 (s, 3 H, 1 C*H_3_*); ^13^C-NMR (CDCl_3_): δ 159.9 (Cq_ArOMe_), 136.7 (Cq_ArSPh_), 130.1 (Cq_ArCH2O_), 130.1 (C_Ar_), 129.9 (C_Ar_), 129.5 (C_Ar_), 126.7 (C_Ar/paraS_), 114.3 (C_Ar/orthoOMe_), 109.2 (Cq isopropylidene), 78.0 (C-2), 77.8 (C-4), 77.6 (C-3), 73.3 (*C*H_2_Ph), 70.0 (C-5), 65.1 (C-6), 55.7 (O*Me*), 35.3 (C-1), 27.4 (CH_3_ isopropylidene), 25.5 (CH_3_ isopropylidene); IR (neat) *v*_max_: 3422, 2987, 2933, 1612, 1514, 1249, 1070, 1035, 744 cm^–1^; HRMS (*m/z*, ESI) calculated for C_23_H_30_O_6_NaS: (M+Na) = 457.1661 (calculated), 457.1649 (found). 

Minor diol (*3,4-di-O-isopropylidene-2-O-(4-methoxybenzyl)-1-S-phenylthio-D-galactitol*): ^1^H-NMR (CDCl_3_): δ 7.23-7.45 (m, 5 H, H_Ar_), 7.19 (d, 2 H, *J*_ortho,meta/OMe_ 8.6 Hz, H_ ortho,meta/OMe_), 6.84 (m, 2 H, H_ meta,ortho/OMe_), 4.65 (d, 1 H, *J*_CHPh,CPh_ 11.0 Hz, C*H*_2_Ph), 4.52 1 H, (dd, *J*_3,4_ 6.6 Hz, *J*_3,2_ 2.0 Hz, H-3), 4.40 (d, 1 H, C*H*_2_Ph), 4.02 (d, 1 H, H-4), 3,80 (s, 3 H, OMe), 3.68–3.76 (m, 1 H, H-2 or H-5), 3.61–3.68 (m, 1 H, H-2 or H-5), 3.56 (s, 1 H, OH), 3.50 (s, 2 H, H-6), 3.35 (dd, 1 H, *J*_1a,2_ 4.1 Hz, *J*_1a,1b_ 13.7 Hz, H-1a), 3.22 (dd, 1 H, *J*_1a,2 _8.9 Hz, H-1b), 1.51 (s, 3 H, 1 C*H_3_*), 1.27 (s, 3 H, 1 C*H_3_*).

*6-O-(tert-Butyldimethylsilyloxy)-3,4-di-O-isopropylidene-2-O-(4-methoxybenzyl)-1-S-phenylthio-L-altritol* (**14**)


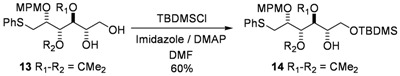


Imidazole (28 mg, 0.4 mmol), TBDMSCl (67 mg, 0.44 mmol) and DMAP (10 mg, 80 mmol) were added to a solution of **13** (175 mg, 0.4 mmol) in dry DMF (4 mL) at 0 °C. After warming to room temperature and stirring for 6 h, the reaction mixture was diluted with CH_2_Cl_2_ (50 mL), washed with water (5 mL), and then with saturated NH_4_Cl (5 mL). The organic phase was dried with MgSO_4_, filtered and evaporated, and the residue purified by column chromatography to give **14** (130 mg, 60%) as a colorless oil. The crude alcohol **14** was used without any further purification.

*6-O-(tert-Butyldimethylsilyloxy)-3,4-di-O-isopropylidene-2-O-(4-methoxybenzyl)-5-O-(R)-2-methoxy-phenylacetyl-1-S-phenylthio-L-altritol *(**15**) *and 6-O-(tert-Butyldimethylsilyloxy)-3,4-di-O-isopropyl-idene-2-O-(4-methoxybenzyl)-5-O-(S)-2-methoxyphenylacetyl-1-S-phenylthio-L-altritol* (**16**)


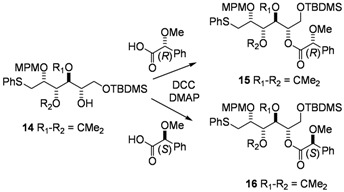


DMAP (3 mg, 20 mmol), (−)-*R*-MPA (80 mg, 0.48 mmol) and DCC (99 mg, 0.48 mmol) were added to a solution of **14** (65 mg, 120 mmol) in dry CH_2_Cl_2_ (5 mL) at 0 °C. After stirring for 1 h, the reaction mixture was diluted with CH_2_Cl_2_ (30 mL), washed with saturated NH_4_Cl (5 mL), and then with brine (5 mL). The organic phase was dried with MgSO_4_, filtered and evaporated. The residue was purified by column chromatography to give **15** as a colorless oil. ^1^H-NMR (CDCl_3_): δ 7.13–7.46 (m, 12 H, H_Ar_), 6.85 (d, 2 H, *J*_ortho,meta/OMe_ 8.6 Hz, H_Ar_), 5.17 (m, 1 H, *J*_5,4_ 8.0 Hz H-5), 4.63 (s, 3 H, C*H*OMe), 4.58 (d, 1 H, *J*_CHPh,CPh_ 11.1 Hz, C*H*_2_Ph), 4.50 (d, 1 H, C*H*_2_Ph), 4.47 (m, 2 H, H-3, H-4), 3.80 (s, 3 H, OMe), 3.73 (m, 2 H, *2 *H-6), 3.44 (m, 1 H, H-2), 3.29 (m, 3 H, CHO*Me*), 3.23 (m, 2 H, *2 *H-1), 1.46 (s, 3 H, 1 C*H_3_*), 1.34 (s, 3 H, 1 C*H_3_*), 0.77 (s, 9 H, 1 C(C*H_3_*)*_3_*), −0.11 (s, 3 H, SiC*H_3_*); −0.17 (2s, 2 × 3 H, 2 SiC*H_3_*). The same procedure with (+) *S*-MPA gave **16** as a colorless oil. ^1^H-NMR (CDCl_3_): δ 7.11–7.47 (m, 12 H, H_Ar_), 6.82 (d, 2 H, *J*_ortho,meta/OMe _8.6 Hz, H_Ar_), 5.20 (m, 1 H, *J*_5,4_ 7.2 Hz H-5), 4.69 (s, 3 H, C*H*OMe), 4.52 (d, 1 H, *J*_CHPh,CPh_ 11.0 Hz, C*H*_2_Ph), 4.37 (d, 1 H, C*H*_2_Ph), 4.34 (dd, 1 H, *J*_4,3_ 6.2 Hz, H-4), 4.03 (dd, 1 H, *J*_3,2_ 1.2 Hz, H-3), 3.91 (dd, 1 H, *J*_6a,6b_ 11.5 Hz, *J*_6a,5_ 1.5 Hz, H-6a), 3.78 (s, 3 H, OMe), 3.76 (dd, 1 H, *J*_6b,5_ 4.4 Hz, H-6b), 3.35 (m, 3 H, CHO*Me*), 3.12 (m, 1 H, H-2), 3.00 (dd, 1 H, *J*_1a,2 _4.5 Hz, *J*_1a,1b _12.7 Hz, H-1a), 2.94 (dd, 1 H, H-1b), 1.41 (s, 3 H, 1 C*H_3_*), 1.28 (s, 3 H, 1 C*H_3_*), 0.87 (s, 9 H, 1 C(C*H_3_*)*_3_*), 0.01 (2s, 2 × 3 H, 2 SiC*H_3_*).

*1,2-Anhydro-3,4-di-O-isopropylidene-2-O-(4-methoxybenzyl)-1-S-phenylthio-L-altritol* (**7**)


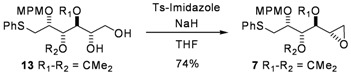


Sodium hydride 60% suspension in mineral oil (56 mg, 1.7 mmol) was added to a solution of **13** (100 mg, 0.23 mmol) in dry THF (2.4 mL) at 0 °C After 15 min at 0 °C tosylimidazole (104 mg, 468 mmol) was then added. The reaction mixture was stirred for 16 h. After addition of diethylether (20 mL), washing with saturated sodium bicarbonate (5 mL), saturated ammonium chloride (5 mL) and brine (5 mL), the combined organic phases were dried with MgSO_4_, filtered and evaporated, and the residue purified by column chromatography to give **7** (71 mg, 74%) as a slightly yellow oil. [α]^20^_D_ = −16.4° (C = 0.22, CHCl_3_); ^1^H-NMR (CDCl_3_): δ 7.43 (d, 2 H, *J*_meta,ortho_ 7.7 Hz, H_Ar_), 7. 3 (m, 4 H, H_Ar_), 7.21 (m, 1 H, H_Ar_), 6.87 (d, 2 H, *J*_ortho_ 8.5 Hz, H_Ar_), 4.67 (d, 1 H, *J*_gem_ 11.0 Hz, C*H*_2_Ph), 4.64 (d, 1 H, *J*_gem_ 11.0 Hz, C*H*_2_Ph), 4.52 (dd, 1 H, *J*_3,4_ 5.7 Hz, *J*_3,2_ 5.6 Hz, H-3), 3.96 (ddd, 1 H, *J*_2,1_ 5.6 Hz, H-2), 3.82 (s, 3 H, OMe), 3.59 (dd, 1 H, *J*_4,5_ 8.1 Hz, H-4), 3.35 (dd, 1 H, *J*_gem_ 13.6 Hz, H-1a), 3.25 (dd, 1 H, H-1b), 3.12 (ddd, *J*_5,6a_ 4.9 Hz, *J*_5,6b_ 2.5 Hz, 1 H, H-5), 2.83 (dd, 1 H, H-6a), 2.62 (dd, 1 H, H-6b), 1.53 (s, 3 H, 1 C*H_3_*), 1.36 (s, 3 H, 1 C*H_3_*); ^13^C-NMR (CDCl_3_): δ 159.2 (Cq_ArOMe_), 136.3 (Cq_ArSPh_), 130.4 (Cq_ArCH2O_), 129.6 (2C_Ar_), 128.9 (C_Ar_), 126.2 (C_Ar/paraS_), 113.7 (C_Ar/orthoOMe_), 109.2 (Cq isopropylidene), 78.8 (C-4), 78.7 (C-3), 76.3 (C-2), 72.3 (*C*H_2_Ph), s55.3 (O*Me*), 49.4 (C-5), 46.1 (C-6), 35.1 (C-1), 27.6 (CH_3_ isopropylidene), 25.3 (CH_3_ isopropylidene); IR (neat) *v*_max_: 3057, 2989, 2933, 1653, 1615, 1559, 1540, 1515, 1248, 1086, 1038, 741 cm^–1^; HRMS (*m/z*, ESI) calculated for C_23_H_28_O_5_NaS: (M+Na) = 439.1555 (calculated), 439.1557 (found).

## 4. Conclusions

We have developed a short and efficient procedure for the synthesis of a diversely protected rare L-altritol derivative **7** (which could also be seen as a L-talitol derivative). This key compound offers access to the central stereoclusters of the sagittamide backbones. Work is in progress to extend this efficient pathway to making useful fragments in order to definitively establish the exact configurations of these natural substances. 
